# Green Synthesis of Biocompatible Chiral Gold Nanoparticles

**DOI:** 10.3390/polym16233333

**Published:** 2024-11-28

**Authors:** Yuan Fan, Na Li, Jiaolong Wang, Lan Liao, Junchao Wei

**Affiliations:** 1 School of Stomatology, Jiangxi Medical College, Nanchang University, Nanchang 330006, China; fanyuan51888@163.com (Y.F.); wangjl6687@163.com (J.W.); 2 Jiangxi Province Key Laboratory of Oral Disease, Nanchang 330006, China; pingguonana1999@163.com; 3 Jiangxi Province Clinical Research Center for Oral Disease, Nanchang 330006, China

**Keywords:** alginate, chiral, gold, nanoparticle, green synthesis

## Abstract

Chiral gold nanoparticles (Au NPs) have been investigated widely and have shown great potential in biomedical applications, such as biosensing, combating bacterial infections and tissue regeneration. However, some stabilizers and reducing agents for the synthesis of chiral Au NPs can produce toxicity in living organisms. Therefore, it is interesting to design green methods to prepare chiral gold nanoparticles that are nontoxic, environment-friendly, and low-cost. Herein, novel biocompatible chiral Au NPs with a diameter of 54.4 ± 14.9 nm were prepared by the in situ reduction of HAuCl_4_ with alginates as the green reducing agent and chiral-inducing and stabilizing agent. XPS, TGA, UV-Vis and CD analyses demonstrated that alginate-stabilized chiral Au NPs (ALG-Au NPs) were successfully prepared, while biocompatibility assessment showed that cell viability was 116.0% when the concentration of ALG-Au NPs arrived at 300 μg/mL, which indicated that ALG-AuNPs showed excellent biocompatibility. Furthermore, the ALG-Au NPs can respond to metal ions, such as Ca^2+^, Cu^2+^, Mn^2+^ and so on, implying potential application for biosensing.

## 1. Introduction

Chirality is a universal phenomenon in nature and plays significant roles in different lengths of scale, from small molecules to biological systems and then to galaxies [1]. Chiral substances widely exist in biological systems, such as DNA, amino acids and peptides, and these chiral macromolecules are crucial for normal physiological activities [2]. For example, amino acids are always levorotatory (L), whereas glucose is always dextrorotatory (D) in nature and living organisms. Furthermore, L- and D-configuration can have different effects on living things, for example, D-thalidomide can alleviate pregnancy reactions, while L-thalidomide presents teratogenicity. Due to the special biological performance of chirality, many researchers have desired to extend chirality to inorganic nanomaterials to broaden the biological application of nanomaterials [3,4]. Different from natural chiral substances, inorganic nanomaterials show a large surface-to-volume ratio, surface plasmon resonance and quantum confinement effect [5]. When inorganic nanoparticles (NPs) are endowed with chirality, they display new characteristics, such as a high g-factor and enantiomeric configuration [6]. Therefore, inspired by the unique properties of chirality, it is interesting and important to investigate the preparation and properties of chiral inorganic nanomaterials.

In the recent decades, chiral inorganic nanomaterials, that is, nanoparticles associated with the chirality of surface ligands, the shape of inorganic cores, the chiral reconstruction of nanoparticle surfaces and the symmetry breaking of the geometry, have been widely investigated. Various chiral inorganic nanoparticles, such as chiral gold (Au), ZnO and Ag NPs, have shown great potential in biosensing, biotherapy, catalysis, medicine and so on [7]. Therefore, the preparation of chiral inorganic nanomaterials has aroused much attention and been an attractive field [8]. Chiral gold nanoparticles have been widely investigated due to their morphological diversity, high stability, surface functionalization and plasmatic properties [9]. Up until now, many chemical methods have been used to prepare chiral Au NPs [10]. For example, Kotov’s group prepared many chiral Au nanomaterials with NaBH_4_/ascorbic acid/citrate as the reducing agent and cetyltrimethylammonium bromide (CTAB) as the stabilizer by the seed-mediated growth method [11,12,13]. Moreover, Kimura et al. used the HAuCl_4_, L/D-penicillamine, NaBH_4_ in water–toluene dual phase solution to prepare chiral gold clusters [14]. However, most of the chemical methods for the synthesis of chiral Au NPs involve some harmful reagents such as CTAB [11], sodium borohydride [13], trioctylphosphine oxide [15], toluene and chloroform [16], which results in potential environmental toxicity or biotoxicity and thus limits the further biological applications of chiral Au NPs. Therefore, the green synthesis of chiral Au NPs via nontoxic raw materials, such as natural molecules, plant extracts or microorganisms, has attracted great attention due to its biocompatible, environment-friendly, and low-cost features [17,18].

Alginates are chiral natural polysaccharides derived from brown seaweed, which are composed of (1-4)-linked β-D-mannuronic acid (M-blocks) and α-L-guluronic acid (G-blocks) saccharide residues [19]. Due to its biocompatibility and biodegradability, ALG has been widely used in biomedical fields. In addition, ALG is a hydrophilic polymer and possesses multi-functional groups; therefore, ALG has been widely used as a colloidal stabilizer to prepare and prevent the aggregation of NPs [20,21]. For example, ALG-stabilized silver nanoparticles were prepared via ascorbic acid as the reducing agent and showed good antibacterial properties [22]. In addition, the microwave-assisted green synthesis of metallic nanoparticles including silver and Au NPs with sodium alginate as the reducing and stabilizer agent was reported [23]; the method is simple and highly efficient to prepare metal nanoparticles. However, in this method, the microwave is a key and indispensable condition to reduce the metal ions. Moreover, methods of microwave-assisted green synthesis of Au NPs with alginate as the stabilizer have been comprehensively reported, and the main reason for the reduction of AuCl_4_^−1^ ions into Au NPs is due to highly reactive species, such as hydrogen and hydroxyl radicals, which are decomposed from H_2_O molecules under the microwave-induced plasma in liquid [24]. As a matter of fact, alginate itself can work as a reducing agent; besides, alginates are chiral molecules, which may be used to induce the formation of chiral nanomaterials and thus their application in reducing metal ions and their effect on the chiral optical properties and applications of metal particles are also an interesting topic to be investigated [25,26].

In this work, alginate was used as a reducing agent to prepare chiral Au NPs (ALG-Au NPs), whose chirality resulted from the chirality of surface ligands and the arrangement of surface atoms. As well, alginate also worked as the stabilizer agent and connected on the surface of Au NPs, which made the NPs disperse well in aqueous solution and showed excellent biocompatibility. Furthermore, due to the abundant active groups of ALG, when ALG-Au NPs interacted with metal ions such as Ca^2+^, Cu^2+^ and Mn^2+^, the optical signal changed in the UV-Visible spectrum, implying that ALG-Au NPs can respond to multi-metal ions and show potential for biosensing. Notably, alginate as a natural polysaccharide shows chiral properties, and these made the ALG-Au NPs show a chiral signal, which may have many desirable properties and applications in future.

## 2. Materials and Methods

### 2.1. Materials

Chloroauric acid tetrahydrate (HAuCl_4_·4H_2_O) was purchased (Sinopharm Chemical Reagent Co., Ltd., Shanghai, China). Sodium alginate (98%) was purchased (Aladdin Scientific Co., Ltd., Shanghai, China). 3-(4,5-dimethylthiazol-2-yl)-2,5-diphenyltetrazolium bromide (MTT) was purchased (Beyotime Biotechnology Co., Ltd., Shanghai, China). Acridine orange/ethidium bromide (AO/EB) was purchased (BestBio Co., Ltd., Shanghai, China) Phosphate-buffered saline (PBS) solution was purchased (Solarbio Science & Technology Co., Ltd., Beijing, China).

### 2.2. Preparation of ALG-Au NPs

**Scheme 1:**
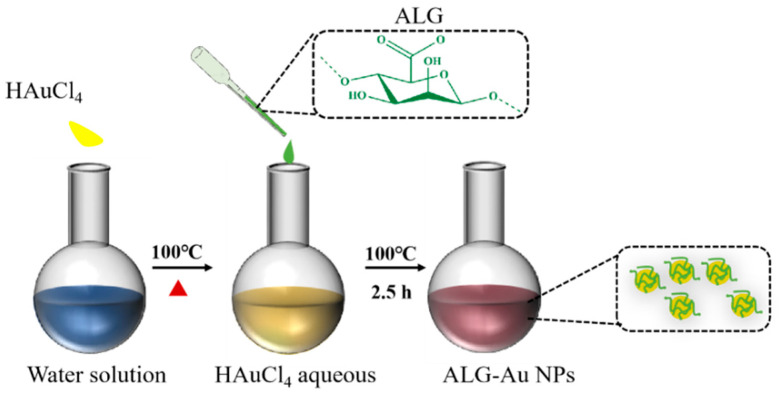
Schematic illustration of chemical procedures for synthesizing ALG-Au NPs.

ALG-Au NPs were prepared by in situ reduction of HAuCl_4_·4H_2_O via sodium alginate as the reducing and stabilizer agents (Scheme 1). Briefly, 50 mL of HAuCl_4_·4H_2_O aqueous solution (1 mg/mL) was added into a 250 mL round-bottomed flask and heated to boiling, then 10 mL sodium alginate aqueous solution (10 mg/mL) was dropped into the HAuCl_4_·4H_2_O solution and stirred till the color of the solution changed from yellow to deep pink.

### 2.3. Characterization of ALG-Au NPs

Elemental analysis of samples was performed by X-ray photoelectron spectroscopy (XPS, Thermo Fisher Scientific, ESCALAB 250Xi, Bend, OR, USA). The morphology and structure of ALG-Au NPs were observed by transmission electron microscopy (TEM, JEOL, JEM-2100, Tokyo, Japan). The corresponding size distribution histograms of ALG-Au NPs was calculated by Image J software (v1.8.0) from many TEM images. The weight-loss analysis of samples was conducted by thermal gravimetric analyzer (TGA, STA449C, Netzsch, Selb, Germany). The zeta potential of ALG-Au NPs was measured by Zetasizer (Zeta, 90Plus PALS, Brookhaven, NY, USA).

### 2.4. Optical Properties of ALG-Au NPs

The chiral optical properties of ALG-Au NPs were measured via circular dichroism spectrometer (CD, J-1500, JASCO, Hachioji, Tokyo). The UV-Vis spectrum of samples was obtained with an Ultraviolet−Visible spectrometer (UV-Vis, 50-coin, Thermo Fisher Scientific, Bend, OR, USA).

### 2.5. Biocompatibility Assessment

The MTT and living/dead staining methods were used to evaluate the biocompatibility of ALG-Au NPs. Firstly, ALG-Au NPs were soaked in cell medium (DMEM, 10% FBS), and the concentrations were set to 37.5, 75, 150, 300 μg/mL, respectively, and placed in a 37 °C incubator for 2 days. Afterwards, leaching solution was obtained by filtration and used for cell viability assays.

The living/dead staining method was carried out with an AO/EB Stain Kit. The cells were seeded in a 96-well plate at a density of 2 × 10^4^ cells/mL and cultured for 24 h. Then, the cell medium was replaced with leaching solution. After 24 h co-culture, 10 μL AO/EB agent was added to the 96-well plate for 30 s. Images were taken by inverted fluorescence microscope.

The L929 cells at a density of 5 × 10^4^ cells/mL were seeded in a 96-well plate and cultured under 5% CO_2_ atmosphere at 37 °C for 24 h. Then, the cell medium was replaced by leaching solution. After 24 h, the cell medium was removed and 100 μL MTT agent (5 mg/mL, PBS) was added into each well to incubate for 4 h. Subsequently, the MTT agent was removed, and 150 μL dimethyl sulfoxide was added to dissolve the formazan. Finally, the absorbance was measured by a microplate reader at 570 nm.

### 2.6. Optical Response of ALG-Au NPs to Metal Ions

An Ultraviolet−Visible spectrometer (UV-Vis, 50-coin, Thermo Fisher Scientific, Bend, OR, USA) was used to observe the optical response of ALG-Au NPs to metal ions. Briefly, 540 μL of ALG-Au NPs aqueous solution was added to a quartz cuvette, then 60 μL of each metal ion solution (36 mM) was added to form a mixed solution for absorbance measurement.

## 3. Results and Discussion

### 3.1. Characterization

**Figure 1:**
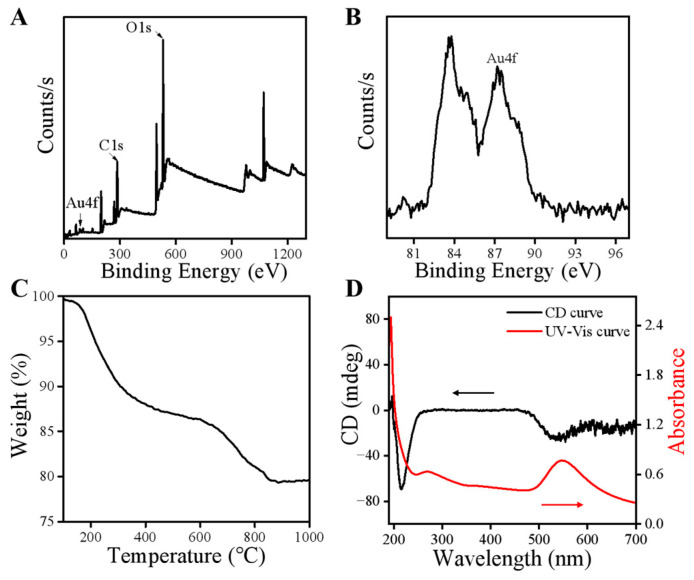
XPS spectra (**A**,**B**), TGA profiles (**C**) and UV-Vis and CD spectra (**D**) of ALG-Au NPs.

To demonstrate the successful preparation of ALG-Au NPs, XPS was used to analyze the surface elements of the samples. As shown in Figure 1A, the XPS curve of ALG-Au NPs showed the characteristic peaks of C_1s_ and O_1s_ at 297 and 521 ev, respectively, which are derived from the alginate molecules. The characteristic peak of Au_4f_ was at 87 ev (Figure 1B), and the atomic percentage of Au_4f_ was much lower than that of C_1s_ and O_1s_, which indicated that many polysaccharide molecules were coated on the surface of the Au NPs. The TGA curve showed that weight loss in ALG-Au NPs mainly occurs from room temperature to 400 °C, derived from the thermal decomposition of ALG, resulting in about 13% weight loss (Figure 1C), which indicated that organic ALG coatings were formed on the surface of Au NPs.

UV-Vis and CD spectrometer analyses were performed to investigate the optical properties of ALG-Au NPs. As shown in Figure 1D, ALG-Au NPs possessed a broad absorption at 550 nm and strong absorption at 193 nm. In the UV range, the characteristic CD peak of ALG-Au NPs at 210 nm was present, which was derived from the chirality of the ALG molecules. Interestingly, a new CD peak emerged at 550 nm for ALG-Au NPs, which may be ascribed to the chiral signal of Au NPs resulting from the interactions between alginates and the Au NPs’ surface. All these results demonstrated that alginates can be successfully used as a reducing agent and a stabilizer to prepare chiral Au NPs with evident plasmonic properties.

**Figure 2:**
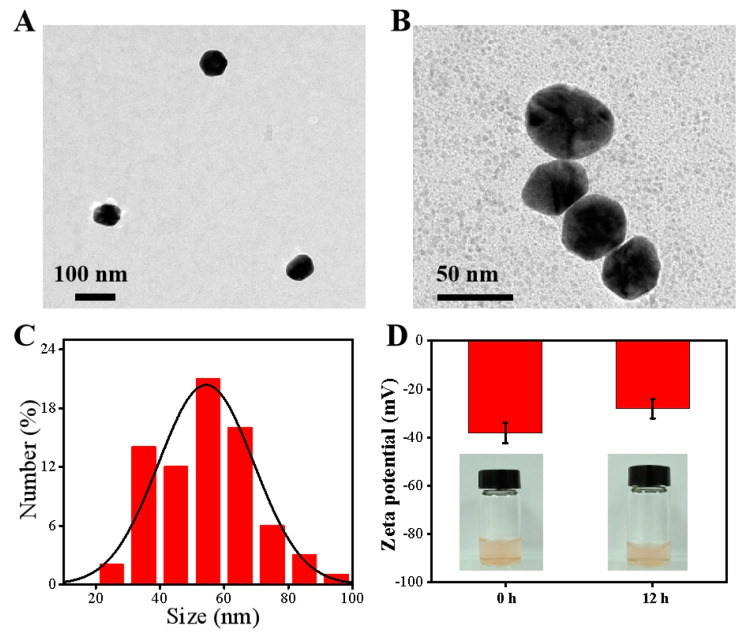
TEM images (**A**,**B**), corresponding size distribution histograms (**C**) and zeta potential and dispersion pictures of ALG-Au NPs (**D**).

TEM images (Figure 2A,B) showed that ALG-Au NPs possessed uniform morphology; from the TEM images, the ALG-Au NPs dispersed very well and no aggregation was found. This may occur because the ALG molecules on the surface of NPs can prevent the aggregation of nanoparticles. Furthermore, based on the TEM images, the corresponding particle size distribution was measured by Image J software (v1.8.0), and the results indicated that the diameter of ALG-Au NPs was about 54.4 ± 14.9 nm (Figure 2C). The zeta potential of ALG-Au NPs was measured dynamically. In the beginning, the zeta potential was about 38.1 mV, 12 h, and the value was 28.1 mV (Figure 2D). This may due to the slightly aggregate nature of the ALG-Au NPs. However, the disperse images showed that the ALG-Au NPs maintained good colloidal stability (Figure 2D), implying that the NPs dispersed well in water, which is accordant with the TEM results.

### 3.2. Biocompatibility of ALG-Au NPs

**Figure 3:**
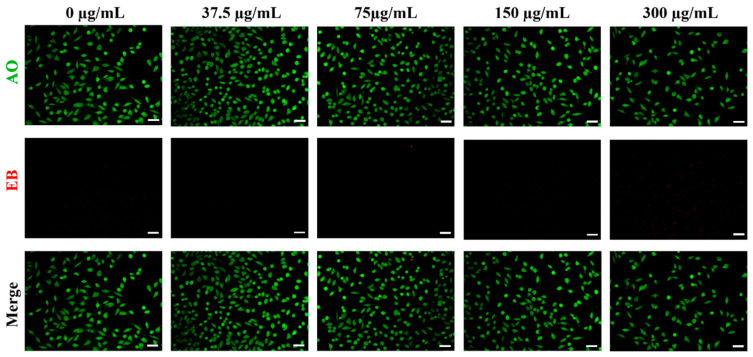
Living/dead staining with AO (green) for live cells and EB (red) for dead and apoptotic cells cultured with ALG-Au NPs at different concentrations (scale bar = 50 μm).

**Figure 4:**
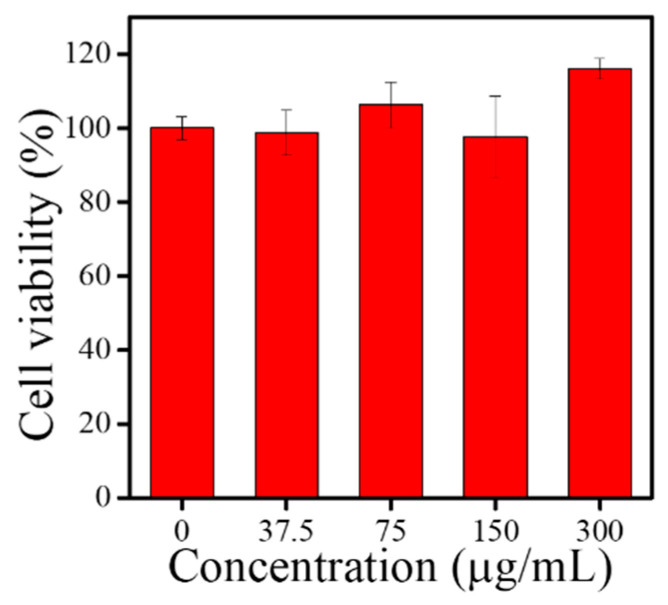
Cell viability of L929 cells treated with ALG-Au NPs at different concentrations.

Biocompatibility is the crucial evaluation criterion of biological materials, which is related to the preparation method of materials, the property of materials and so on. As shown in Figure 3, the results of the AO/EB staining assay found that the cells incubated with ALG-Au NPs at different concentrations showed few dead cells (red fluorescence), which indicated that ALG-Au NPs possessed few toxicities to cells. Furthermore, the results of the MTT assay demonstrated that ALG-Au NPs displayed excellent biocompatibility. When the concentration of ALG-Au NPs was 37.5 μg/mL, the cell viability was 98.7%. When the concentration increased to 75 and 150 μg/mL, the survival rate of the cells was 106.3% and 97.7%, respectively. Further, when the concentration of ALG-Au NPs was 300 μg/mL, the cell viability was 116.0%, which were much higher than that of the control group (Figure 4). Green synthesis of chiral Au NPs is regarded as the ideal strategy due to its easy synthesis and in vivo safety [27]. In this work, ALG-Au NPs showed several advantages over chiral Au NPs synthesized using traditional chemical methods, for example, green solvent, biocompatible reducing agent and no additional steps for removing toxicity surfactants. Thus, all results demonstrated that ALG-Au NPs displayed excellent biocompatibility and could promote the proliferation of cells.

### 3.3. Optical Response of the ALG-Au NPs to Various Metal Ions

**Figure 5:**
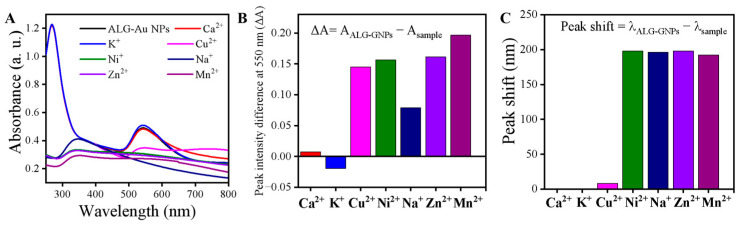
Absorbance spectra (**A**), variations in the absorbance intensity (**B**) and change in absorbance peak shifts (**C**) of ALG-Au NPs reacted with various metal ions. A_ALG-Au NPs_ and λ_ALG-Au NPs_ are the absorbance intensity value and wavelength of the ALG-Au NPs dispersion at 550 nm without any interferents, respectively. A_sample_ and λ_sample_ are the absorbance of mixture of the ALG-Au NPs dispersion and each metal ion solution.

The UV-Vis absorbance of the mixtures of ALG-Au NPs and metal ions is shown in Figure 5A. When ALG-Au NPs interacted with different metal ions, respectively, the absorbance intensity and corresponding peak position of maximum absorption were different (Figure 5B,C). When Ca^2+^ was added into the ALG-Au NPs solution, the UV-Vis signal of the mixture solution showed a strong response, while the absorption and peak shift showed nearly no change. After adding K^+^ to the ALG-Au NPs solution, the ΔA value was negative, which showed that the intensity of the characteristic peak at 550 nm increased. However, when ALG-Au NPs interacted with Cu^2+^, the UV-Vis spectrum showed that the absorption intensity of the characteristic peak was much lower than that of ALG-Au NPs, and the peak position was at 534 nm, which happened to blue-shift the band obviously. Furthermore, when each of Ni^2+^, Na^+^, Zn^2+^ and Mn^2+^ was added to ALG-Au NPs solution, respectively, the absorption intensity significantly decreased and the absorbance band blue-shifted to about 344 nm. All results demonstrated that ALG-Au NPs showed different responses to different metal ions, and this may be ascribed to the different binding intensities between metal ions and ALG-Au NPs, respectively, and the different distances between NPs. The ALG-Au NPs showed an interesting LSPR property and chiral optical properties, and the ALG molecules on the surface of Au NPs could bind with different metal ions via the carboxyl and hydroxyl functional groups, which may have resulted in the change in optical signals of ALG-NPs. Therefore, ALG-Au NPs may work as smart sensors to detect the existence of metal ions.

## 4. Conclusions

In this work, biocompatible chiral Au NPs with diameters of 54.4 ± 14.9 nm were prepared with alginates as the reducing agent and stabilizer (ALG-Au NPs). The results of XPS, TGA, UV-Vis and CD analyses all indicated the successful preparation of chiral Au NPs. AO/EB living/dead cells and MTT staining assay results showed that ALG-Au NPs possessed excellent biocompatibility; the cell viability reached 116.0% when the concentration of ALG-Au NPs arrived at 300 μg/mL, which could enable the application of ALG-Au NPs in the biomedical field. Furthermore, the optical signals deriving from the interactions of ALG-Au NPs and metal ions could be measured by UV-Vis spectrometer and showed different responses for different metal ions, implying the potential application of ALG-Au NPs for biosensing. Although this work is just a primary study on the green synthesis and multi-metal ion response of ALG-Au NPs, ALG-Au NPs still show potential in applications in the biomedical filed due to their excellent biological and optical properties.

## Data Availability

The original contributions presented in the study are included in the article, further inquiries can be directed to the corresponding author.
